# Chest Wall Swelling in a Baby Boomer: An Unusual Presentation of Primary Hepatocellular Carcinoma

**DOI:** 10.7759/cureus.18163

**Published:** 2021-09-21

**Authors:** Venkata Vinod Kumar Matli, Amina Dhahri, Venkatasai Boda Eswara, Linda D Green

**Affiliations:** 1 Internal Medicine, Christus Highland Medical Center, Shreveport, USA; 2 Internal Medicine, University of Maryland Regional Capital Medical Center, Largo, USA

**Keywords:** baby boomer, chronic liver disease (cld), chest wall swelling, hepatitis c (hcv), hepatitis-c infection, hepatocellular carcinoma (hcc)

## Abstract

Primary hepatocellular carcinoma (HCC) is one of the most frequently diagnosed cancers in adult men and a leading cause of cancer-related deaths worldwide. It also has an association with patients with hepatitis C-related cirrhosis. HCC usually metastasizes within the liver as well as to the lungs, regional lymph nodes, and adrenal glands, whereas the involvement of the chest wall and thoracic musculoskeletal system are more unusual.

Herein, we report the case of a 58-year-old man who presented with swelling of the right anterolateral lower chest wall. The final diagnosis was primary HCC with distant metastases involving the right anterolateral ribs and left scapula. Such a presentation of extrahepatic HCC of this size and at this site is unique and has never before been reported in the literature. It reinforces the urgency and importance of screening all adults (18 years and above), particularly baby boomers, because three out of 100 have been infected with hepatitis C, at least once in their lifetime. It is also a wake-up call, as the incidence of primary HCC secondary to hepatitis C-associated cirrhosis has doubled, with a resultant increase in mortality.

This HCC-related death might have been prevented if the patient had been screened for hepatitis C virus in his lifetime, as recommended by the American Association for the Study of Liver Diseases. We also discuss the latest developments in the diagnosis and management of HCC.

## Introduction

The incidence of hepatocellular carcinoma (HCC) has doubled and mortality rates have increased in recent decades [1]. Cirrhosis is the leading cause of primary HCC development regardless of etiology; however, the main risk factors associated with HCC in the United States are infections, which are hepatitis B and C, followed by noninfectious causes, such as alcoholic liver cirrhosis, aflatoxin toxicity, and non-alcoholic steatohepatitis.

Data from the literature show that alcohol-related cirrhosis diagnoses and the associated age-adjusted mortality with subsequent development of HCC have declined. Thus, biologically credible culprits responsible for the rising incidence of HCC are hepatitis B and C, particularly in baby boomers, who account for about 75% of US hepatitis-C cases [2].

## Case presentation

A 58-year-old male patient presented with right-sided chest wall swelling that had been increasing in size for three months.

He had no history of fever, night sweats, or jaundice. Patient history was significant for weight loss of about 20 pounds in the last six months. The patient was not even an alcoholic drinker or tobacco smoker. The patient was admitted for a workup of swelling and loss of weight and appetite. On examination, there was a 6 cm×10 cm oval swelling over the right anterolateral mid-chest wall with a smooth surface located lateral to the nipple. The swelling was firm, non-tender, well-defined, and fixed to the chest wall. The overlying skin was normal. No cervical, axillary, inguinal lymph nodes were palpable. Lungs clear to auscultation. S1, S2 heart sounds were heard in normal intensity and no murmurs were heard. Abdominal examination was soft, non-tender, and non-distended without hepatosplenomegaly.

Preliminary labs, including complete blood picture and basic metabolic panel, were essentially normal but the hepatitis panel tested positive for hepatitis C antibody, which led us in the direction of hepatitis C workup, including liver imaging studies. Admission laboratory results also include a hepatitis C viral (HCV) load of 200,000 IU/L, and the remainder of the hepatitis panel was negative. Other results included alanine aminotransferase (ALT) of 52 units/L, aspartate aminotransferase (AST) of 64 units/L, alkaline phosphatase (ALP) of 120 units/L, platelet count of 170, and albumin of 3.3 g/dL.

**Figure 1:**
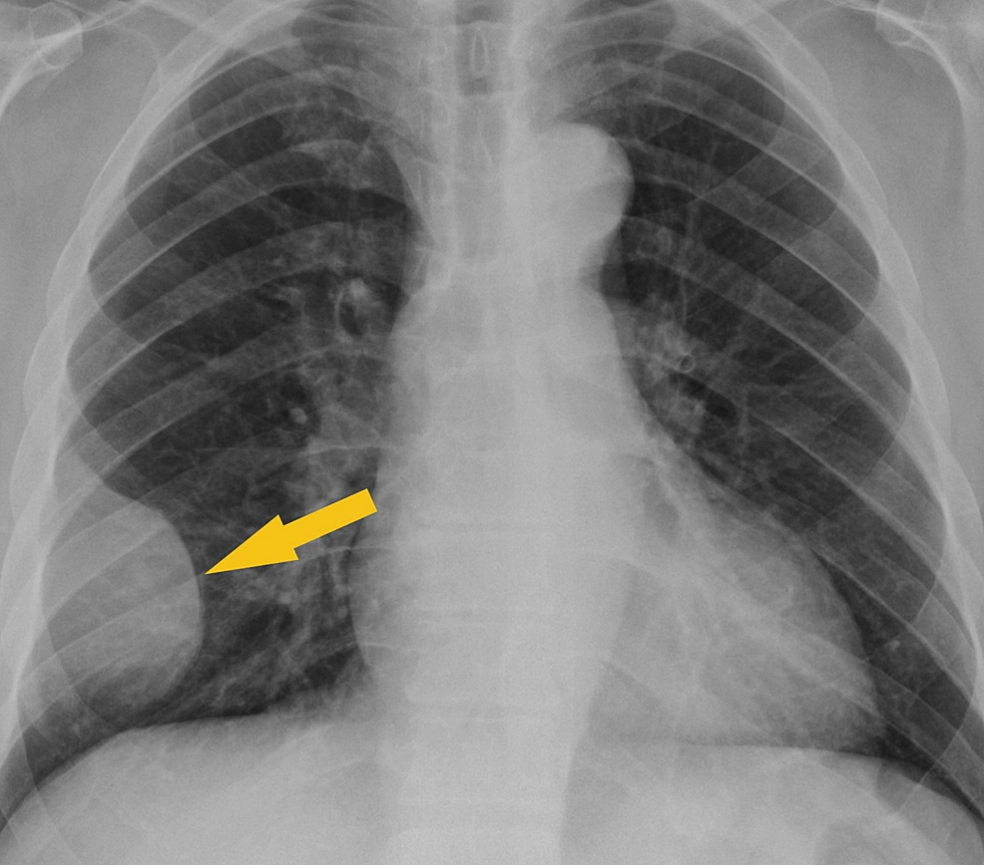
Chest X-ray shows a low-density soft tissue structure located at the periphery of the right hemithorax, measuring approximately 9 cm in size

**Figure 2:**
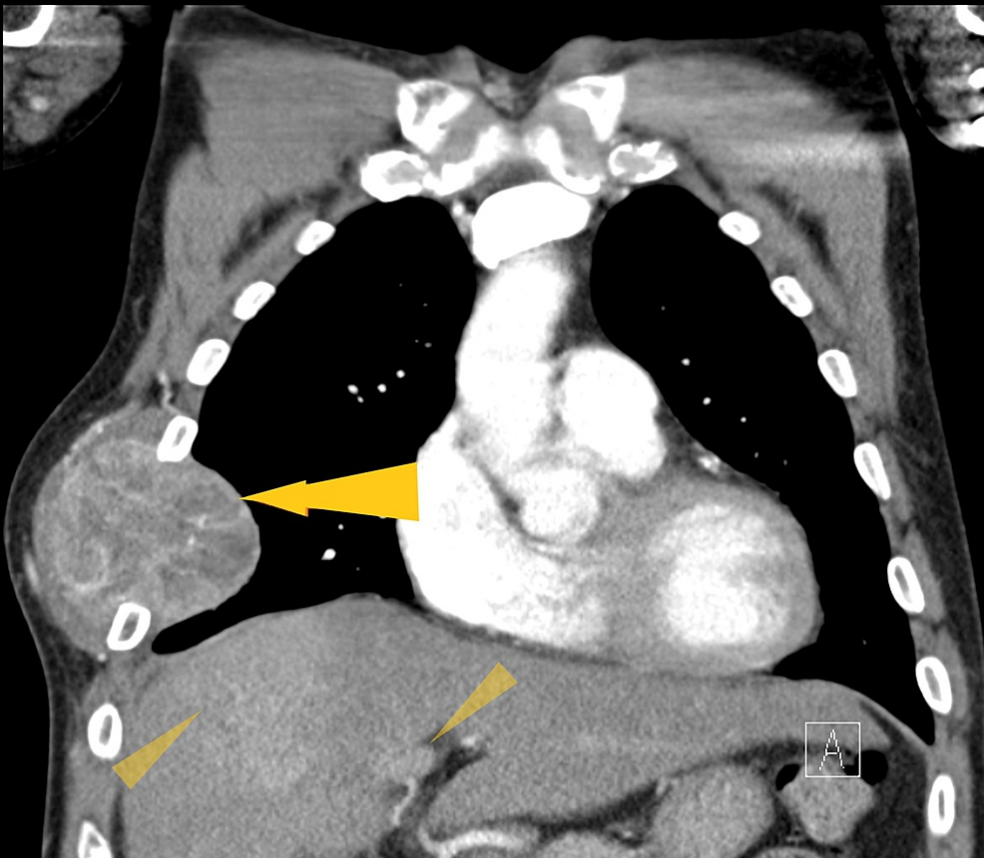
Computed tomography of the chest, with contrast, shows a heterogeneously enhanced soft tissue mass arising from the right anterolateral sixth rib (yellow arrow), which was eroded and replaced by a portion of the mass that appeared extrapleural. Multiple ill-defined, hyper-attenuating foci in the liver are present, including a larger one measuring 2×1.2 cm in the right lobe and one measuring 2.8 cm×1.4 cm in the gastrohepatic lymph node (yellow triangles).

**Figure 3:**
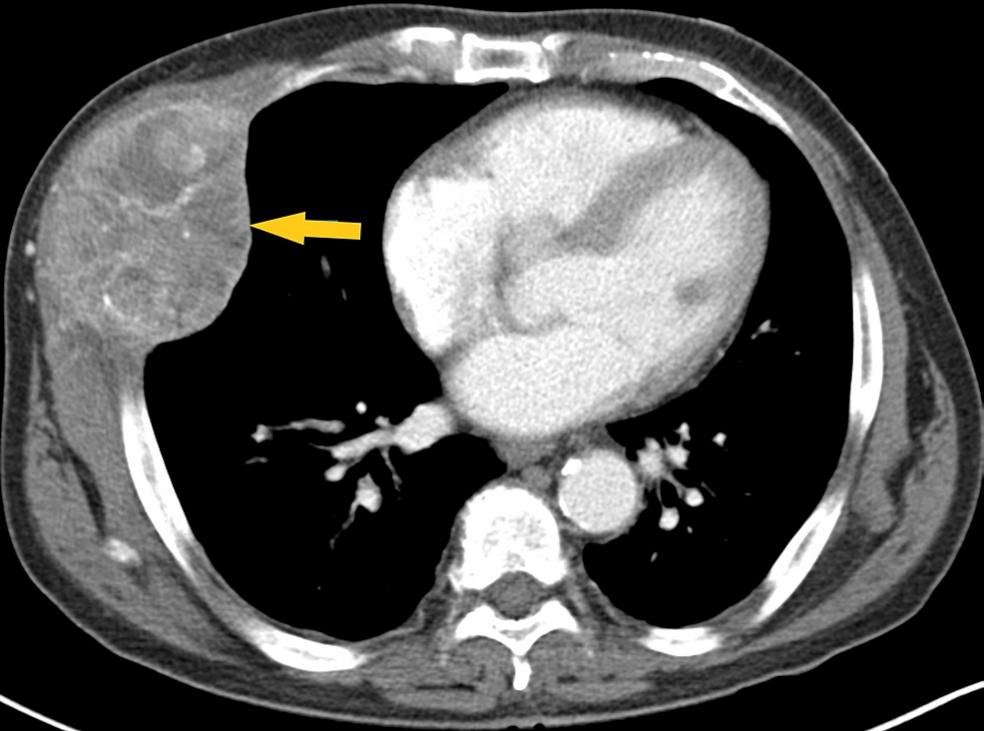
Computed tomography of the chest, with contrast, shows a heterogeneously enhanced soft tissue mass arising from the right anterolateral sixth rib (yellow arrow), which was eroded and replaced by a portion of the mass that appeared extrapleural

**Figure 4:**
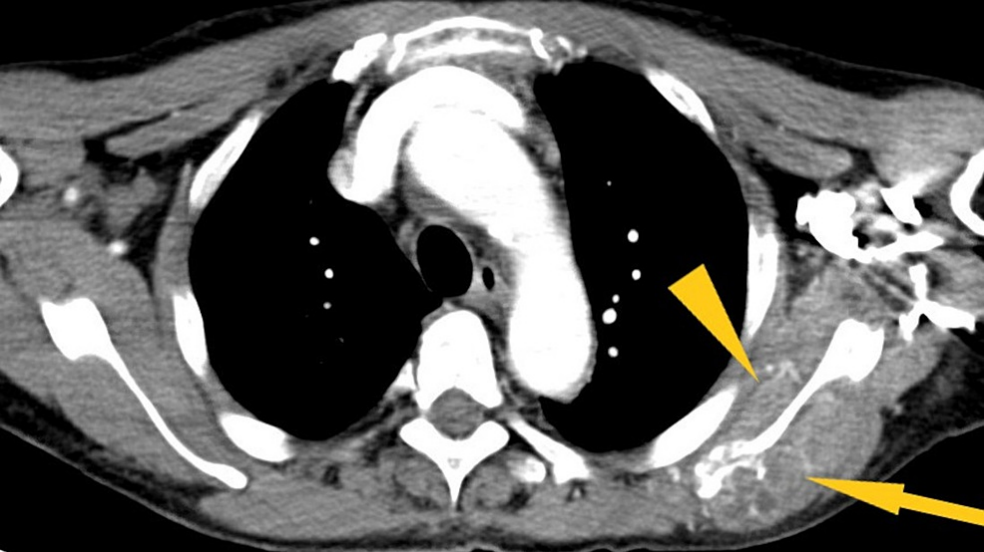
CT chest with contrast shows expansile, extrahepatic hepatocellular carcinoma metastasis to the left scapula (yellow triangle and arrow)

**Figure 5:**
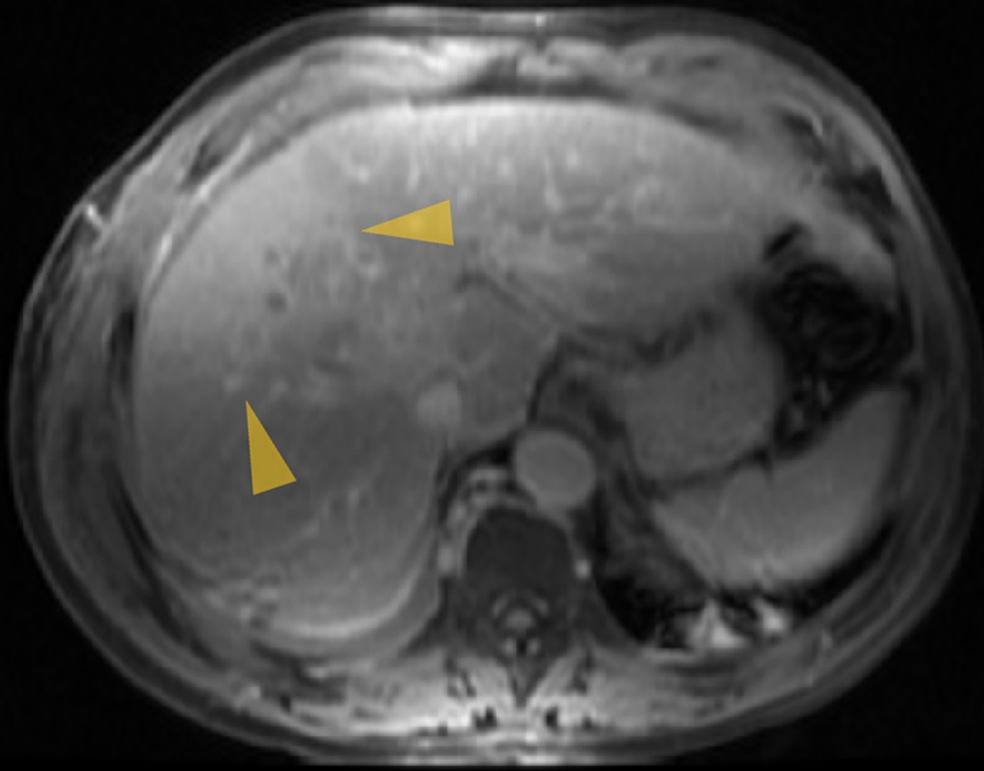
MRI abdomen with contrast showing hypervascular mass lesions with poorly defined margins in both the right and left lobes of the liver. The larger one measures 7 cm×5 cm in the anterolateral surface of the right lobe and abutting the diaphragmatic surface.

**Figure 6:**
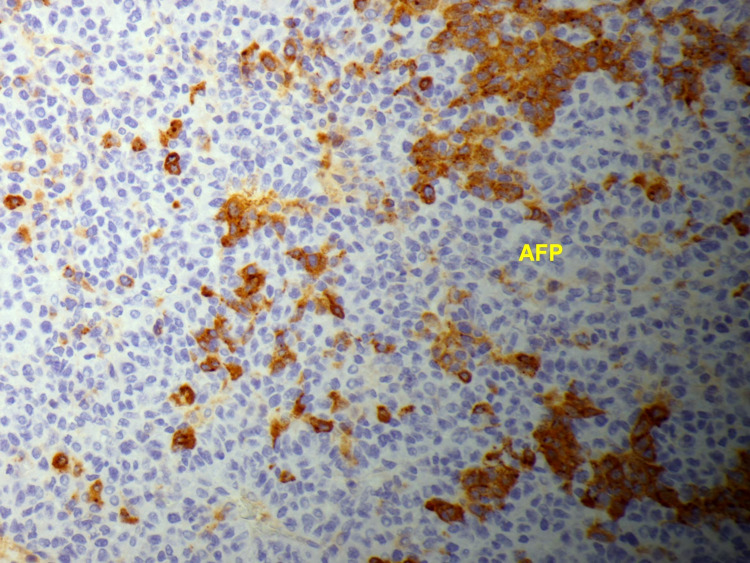
Histopathology with immunohistochemical staining of the biopsy of the chest wall mass shows malignant hepatocytes positive for alpha-fetoprotein, confirming this as a hepatocellular carcinoma metastasis

**Figure 7:**
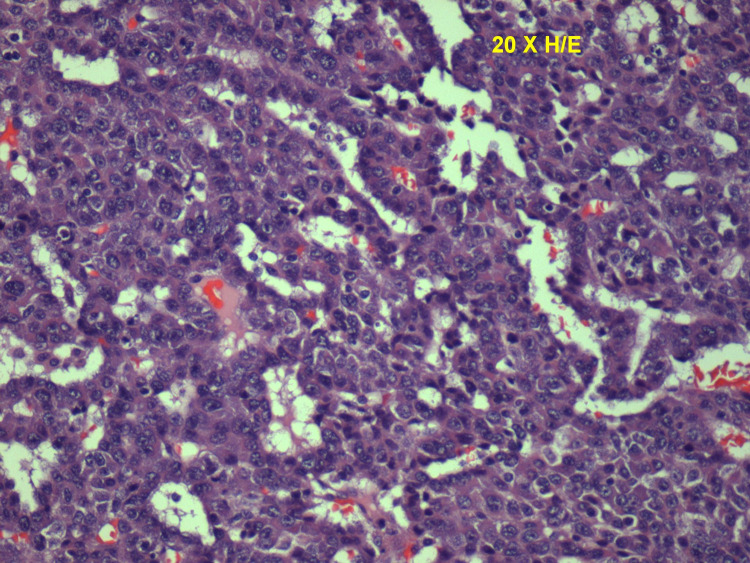
Histopathology slide showing trabecular and sheet-like areas and large malignant cells with high mitotic activity and areas of necrosis

**Figure 8:**
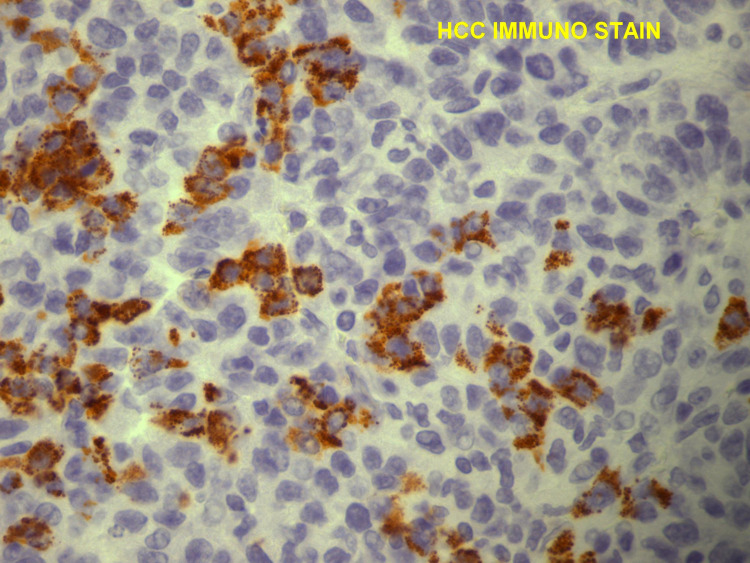
Histopathology with immunohistochemical staining of the biopsy of the chest wall mass shows malignant hepatocytes positive for HCC immunostain, confirming this as a hepatocellular carcinoma metastasis HCC: hepatocellular carcinoma

Chest X-ray (Figure 1) showed a low-density soft tissue structure located at the periphery of the right hemithorax, measuring about 9 cm in size. CT chest with contrast (Figures 2-3) showed a heterogeneously enhanced soft tissue mass arising from the right anterolateral sixth rib, which was eroded and replaced by a portion of the mass that appeared to be extra-pleural in nature. CT chest with contrast (Figure 4) also shows evidence of left scapular metastasis. There were multiple, ill-defined, hyperattenuating foci in the liver, with the largest one measuring 2 cm×1.2 cm in the right lobe. There was also a 2.8 cm×1.4 cm enlarged gastrohepatic lymph node. Abdominal MRI with contrast (Figure 5) showed characteristic features of HCC, including hypervascular mass lesions with poorly defined margins in both the right and left lobes of the liver. The largest one measured 7 cm×5 cm in the anterolateral surface of the right lobe and abutted the diaphragmatic surface. Cytopathological examination of the excisional biopsy of the chest wall mass (Figures 6-7) showed trabecular and sheet-like areas and large malignant cells with high mitotic activity. Immunostaining of tumor cells was done for carcinoembryonic antigen (CEA), carbohydrate antigen (CA 19-9) was negative, but other stains, such as hepatocellular carcinoma antigen (Figure 8) and alpha-fetoprotein (AFP), were positive. Based on the infiltration of these stains and the typical morphological features of tumors, additional immunostains, such as synaptophysin, chromogranin, and S100, were also performed. Tumor cells were positive for synaptophysin. So, the diagnosis of HCC was confirmed. The patient was not a surgical candidate; he underwent palliative treatment with sorafenib and died two years after the date of diagnosis.

## Discussion

HCV accounts for 1% of morbidity and mortality globally [3]. Approximately 2.5% of patients with chronic hepatitis C develop HCC [3]. Baby boomers born between 1946 and 1964 account for 77.3 million people or 25% of the total US population per 2012 census estimates. This is projected to decrease to 19% of the total US population by 2030. Three-quarters of chronic hepatitis C patients belong to the baby boomer group according to prevalence data released by the 2003-2010 National Health and Nutrition Survey [2].

The incidence of HCC is 850,000 cases per year. It remains the second leading cause of cancer-related deaths globally, at approximately 800,000 per year. HCC is the most common primary liver tumor, accounting for 90% of patients [4]. The risk of HCC in cirrhotic hepatitis C patients ranges between 2% and 8% per year, with studies showing a 20-fold increased risk for the development of HCC in HCV antibody-positive patients [5]. Studies from Japan have shown a decreased HCC incidence rate in hepatitis C patients who were treated with anti-HCV agents than in treatment-naïve patients [6-7].

In regard to screening, the American Association for the Study of Liver Diseases (AASLD) released practice guideline recommendations stating that every adult aged 18 years and above should be screened for HCV at least once in their lifetime [8].

We have made remarkable progress in regard to the diagnosis and treatment of HCV with the invention of highly efficacious treatments, with a cure rate of > 95% using anti-HCV antiviral agents with minimal and condonable side effects [9]. We are hoping that at least some parts of the world could be free from hepatitis C because the World Health Organization has issued a global health call to defeat viral hepatitis by 2030 [10].

HCC metastasis occurs most commonly within the liver, and extrahepatic sites often include the lungs, abdominal lymph nodes, and bones [11]. Extrahepatic metastases occur through the lymphatics or hematogenous pathways but rarely through direct spread. The unique presentation in our case study is metastases to the right anterior-anterolateral chest wall involving the ribs and left scapula. The route of spread in our patient appears to be direct spread or through the portosystemic anastomotic vascular channels, which ultimately drain into the intercostal veins via the azygos venous system. Metastasis to the chest wall involving the ribs and left scapula itself is rare; therefore, this pattern is very rare as an initial manifestation of HCC. There are a few case studies reporting HCC presenting as chest wall swelling [12-13], metastases to the pelvic bone, even without a primary lesion [14], intercostal muscles [15], or anterior abdominal wall muscles [16], the right atrium, skeletal muscle, oral cavity, gluteus muscles, and the distal phalanx of the middle finger and the pituitary gland too, but not to the scapula, until now and this is the first case report to show metastasis to the scapula and chest wall with this size. There are case reports of extrahepatic metastases in European literature presenting as ectopic extrahepatic HCC arising in the chest wall without any evidence of primary hepatic HCC on imaging studies; they can arise de novo in ectopic liver tissue [17].

Eighty percent of HCC patients have preexisting cirrhosis, and chronic hepatitis C is the most common etiology after chronic hepatitis B [5]. We believe that remembering the mnemonic A, A, B, and C (A-alcohol, A-aflatoxin, B-hepatitis B, and C-hepatitis C) may be helpful when discussing and studying HCC.

We can divide diagnosis and surveillance into two groups. First, cirrhotic hepatitis C patients do not require invasive procedures, such as liver biopsy, provided the liver lesion on four-phase CT imaging is greater than 2 cm with vascular features suggestive of HCC. These features include arterial phase hyperenhancement and washout, capsule appearance, and threshold growth on multiphase CT or MRI [18]. Alternatively, HCC is also suggested by a serum AFP level of >200 ng/dl. Secondly, liver biopsy is mandatory in non-cirrhotic hepatitis C patients, even if the lesions show characteristic features suggestive of HCC, as discussed above. HCC surveillance should involve ultrasound imaging every six to 12 months with few exceptions. Consider CT or MRI when ultrasound imaging is either inadequate or chances of HCC are highly likely [18].

We did not have difficulty diagnosing HCC in our patient because of an easily accessible chest wall mass for cytopathological examination and characteristic HCC imaging findings. A liver biopsy is not indicated, as our metastatic site yielded the diagnosis of HCC.

Treatment and prognosis of HCC with or without extrahepatic metastasis depend on intrahepatic liver tumor size. Patients can die of liver failure secondary to uncontrolled growth of the liver mass [19]; both prognosis and tailoring of treatment depend on the size of the liver mass but not on the characteristics of the extrahepatic lesions [20]. While there are many treatment options, including multikinase inhibitors, checkpoint inhibitors, and vascular endothelial growth factor (VEGF) inhibitors, available, none of them are curative except liver transplantation in select patients. The AASLD recommends a multidisciplinary approach, which includes hepatologists, pathologists, transplant hepatologists, surgical oncologists, palliative care providers, and diagnostic radiologists; this shows improved clinical outcomes in these patients, which is further improved if managed in places where tumor boards are established [21].

## Conclusions

Our case report is of interest not only because of the involvement of the chest wall and left scapula but also due to the extent and size of the liver metastatic lesion on the chest wall. Our case report reminds us that one should consider extrahepatic HCC in the differentials during the evaluation of a chest wall swelling in chronic hepatitis patients.

Our case report reinforces the necessity of the need for HCV screening in all adults aged 18 years and above at least once in their lifetime, with more emphasis particularly on baby boomers whose HCV prevalence is remarkably higher than in the normal population. This aims to reduce the risk of the hepatitis C virus associated with primary HCC and its resultant morbidity and mortality. This is achievable because of the remarkable progress we have made by inventing highly efficacious anti-HCV antiviral medications, which carry a cure rate of >95% with minimal and condonable side effects. Fortunately, we do not need invasive procedures such as a liver biopsy to diagnose primary HCC if the imaging studies show typical and characteristic findings on CT or MRI of the abdomen, with few exceptions. Liver biopsy is mandatory in non-cirrhotic patients, even if the imaging findings are suggestive of HCC. Our key clinical message relates to the importance of screening all adults aged 18 years and above, particularly baby boomers, for HCV at least once in their lifetime.
